# Exploration of disease variables (DV) in the management of treatment-resistant depression (TRD) in older adults: Results of a Continuous Quality Improvement (CQI) Project

**DOI:** 10.1192/j.eurpsy.2026.10667

**Published:** 2026-07-31

**Authors:** A. S. Luthra

**Affiliations:** Program for Older Adults, Homewood Health Center, Guelph, Ontario, Canada

## Abstract

**Introduction:**

TRD is defined as inadequate response to two adequate (optimal and duration) trials of two different classes of antidepressants. Literature identifies co-morbid *psychiatric illnesses*, *personality factors*, *medical illnesses* and *patient factors* contributory to TRD. Phenotypic manifestations of each subsequent episode of clinical depression changes over its lifetime of expression and so does the propensity to recurrences. They are referred to as *index episodes specifiers (IES)* and *longitudinal course specifiers (LCS),* respectively. Changes in *index episodes* and *longitudinal course* are due to the phenomenon of *episode sensitization* and *kindling,* respectively*. Diurnal variation (DiV)* is another disease variable established in the literature. I have labelled *IEC, LCS and DiV* as DV*.*

**Objectives:**

I hypothesize that identification, and treatment of the DV is the key to increasing the probability of response in established TRD in older adults. The principles governing the project design were derived from the *observational case study* design under the evaluative framework of the *Continuous Quality Improvement* governance structure. Results of CQI project on a Program for Older Adults (POA), a tertiary mood disorders program at Homewood Health, Guelph, Ontario, are presented to support this *a priori hypothesis*

**Methods:**

A random sample of patients admitted to POA were included in the project. Each patient was routinely evaluated with Geriatric Depression Scale (GDS)] and Becks Depressions Inventory (BDI), on admission and at discharge. DSM-IV-R pictogram was used to establish *LCS.* Each patient was evaluated two to three times a week to establish *IES* and *DiV.* Formulated DV were used t0 design pharmacological intervention each week. Each patient also engaged in evidence informed psychotherapies.

**Results:**

100 patients (**70** females: **29** males) were evaluated. The average admission score of the patients on GDS was **20.99** and upon discharge was **6.25** (p<0.00000000000000000000000000000000000000517). The average admission score of the patients on BDI was **26.32** and upon discharge was **6.21** (p<0.00000000000000000000000000000000203). **93** of the 100 patients had complete remission on GDS and **92** of the 100 had complete remission on BDI. **7** of the 100 patients showed partial response on GDS and **7** of the 100 patients showed partial response on BDI. **0** of the 100 patients showed no response on GDS and **1** of 100 patients showed no response on BDI.

**Conclusions:**

Over 90% of the established TRD in older adults responded to the individually tailored pharmacological interventions targeting the DV, while concurrently receiving evidenced informed psychotherapies. These results justify the generation of *a priori* hypothesis for the need for identification, and treatment of DV prior to labelling older adults as TRD.

**Disclosure of Interest:**

None Declared

